# The effect of the preceptorship training program on the participation of clinical nurses in training nursing internship students: a quasi-experimental study

**DOI:** 10.1186/s12912-024-02034-4

**Published:** 2024-06-07

**Authors:** Razieh Mashayekh, Abbas Ebadi, Batool Nehrir, Malihe Sadat Moayed

**Affiliations:** 1 Student Research Committee, Baqiyatallah University of Medical Sciences, Tehran, IR Iran; 2 Nursing Research Center, Clinical Research Institute, Faculty of Nursing, Baqiyatallah University of Medical Sciences, Tehran, Iran

**Keywords:** Education, Instructor, Mentor, Nurses, Cooperative performance, Students

## Abstract

**Introduction:**

Clinical education is a fundamental part of nursing professional education. One method of education is the implementation of the preceptorship program, in which clinical nurses are responsible for educating nursing students. The present study aimed to determine the effectiveness of the preceptorship training program for the participation of clinical nurses in the education of nursing students.

**Methods:**

This quasi-experimental study was conducted in 2023 at the teaching hospital in Tehran, Iran. The sample consisted of 66 nurses from a teaching hospital selected using the accessible method and randomly were put into two groups. The workshop addressed the educational needs of preceptors and students, their readiness for their role, and strategies to effectively support students. Prior to the study, the nurses’ level of participation was assessed utilizing a valid IMSOC questionnaire that gauged their involvement in guiding nursing students. The participation rate was reassessed after one month using the same questionnaire. The data were analyzed using SPSS version 19.

**Findings:**

The two groups were homogeneous in terms of demographic characteristics. The average score for nurses’ participation in the education of students before the intervention was 101.84 ± 15.42 in the test group and 107.24 ± 10.53 in the control group; these two groups were not significantly different (*P* = 0.10). After the intervention, the scores reached 118.90 ± 15.11 in the test group and 106.21 ± 11.96 in the control group, indicating a significant difference (*P* < 0.001). A comparison of the nurses’ participation scores in the test group indicated a significant difference from the pretest to the posttest, with the improvement in all nurses’s participation scores (*P* < 0.001). However, in the control group, this difference was not significant (*P* = 0.41).

**Conclusion:**

The preceptorship training program is effective in light of increasing the participation of clinical nurses in the education of nursing students. This program can improve various aspects, such as motivation, satisfaction, commitment, implementation, and obstacle removal. Considering the importance of clinical training for nursing students and the essential role of preceptors, it is recommended that managers and health trustees in all university hospitals implement a preceptorship training program to increase the participation of clinical nurses in the education of nursing students.

## Introduction

Training human resources in medical sciences is an important task in higher education, as it contributes to the provision, maintenance, and improvement of society’s health [1]. The correct educational process focuses on guiding students toward holistic growth, which is considered a crucial criterion for the functional success of universities [2].

Nurses, as key members of the medical science community, play vital roles in achieving care and treatment goals. In recent decades, nursing education has rapidly developed and expanded within the higher education system worldwide [3]. The position of nursing education holds great importance, as any deficiencies or insufficiencies in education can impact the quality and quantity of health services, ultimately affecting the health of individuals and society [4].

The core of education in medical sciences, particularly in the nursing profession, lies in clinical education. Its evaluation significantly influences the quality of practical education, which is acknowledged by all managers and educational planners [5]. Most of the educational programs mentioned above are clinically oriented, with students spending the majority of their courses in practical settings. Engaging in practical situations while utilizing reasoning, analysis, and decision-making abilities fosters meaningful and lasting learning in students. This is where students translate their theoretical knowledge into practical skills [6, 7].

There are various approaches to clinical education. The change and increase in the number of students as well as the use of skilled and experienced clinical nurses in the education of nursing students under the preceptorship model have opened up new possibilities in clinical learning. The premise of this approach is that working with qualified and experienced nurses will enhance students’ learning [8–10]. Another relevant study revealed that selecting nurse preceptors who possess proficiency and passion for supportive and reflective approaches is vital for cultivating a positive work atmosphere. Additionally, initiatives such as nursing preceptorship help enhance healthcare quality improvement outcomes [11]. Nursing students in longitudinal training have more chances to provide patient care independently and feel more prepared for independent practice by the end of their program. Key factors include long-term relationships with preceptors and the healthcare team as well as fostering professional growth and integration into the team for independent care [12].

The preceptorship educational model is a teaching and learning method in which the preceptor’s goal is to help students develop problem-solving skills and prepare them for various educational, therapeutic, consulting, and managerial roles. This allows undergraduate nursing students to integrate cognitive knowledge with the development of psychomotor and emotional nursing skills [13]. Some studies suggest that this approach is the most effective and appropriate way for nursing interns to acquire competence and skills in dealing with real patients in a clinical setting [14–16]. For example, the final-year operatory room students are satisfied with the new educational model, indicating its effectiveness in clinical education [14]. Furthermore, a review discovered a beneficial effect of utilizing a preceptorship model to enhance clinical instruction for nursing students [15]. Other benefits of the preceptorship model include reducing clinical errors, increasing student satisfaction, fostering greater student cooperation, promoting a sense of safety, and belonging within the nursing group, advocating for student rights, fostering independence, targeting internships, and capitalizing on educational opportunities. Working with competent and experienced nurses ultimately leads to improved student learning [9].

In this regard, in 2021, Dube and colleagues emphasized the use of the model to plan and implement methods that enhance the effectiveness of nursing clinical education [17]. Although the preceptorship includes many of the abovementioned benefits, limitations comprising lack of an expert preceptor in teaching and evaluating students, teaching comprehension as an overhead or stress, conflict, or incompatibility with students should not be ignored [18]. On the one hand, the limited and poor working relationships between hospitals and health education institutions, the lack of patient readiness, and inadequate supervision of professors lead to an unstable clinical environment for students [19, 20]. Studies have shown that most nurses use questionnaires without adequate training in the role of a questionnaire and without knowing how to establish a model [21]. Moreover, the results of a study by Saeedi and colleagues showed that a decrease in the quality of education and associated problems can lead to a decrease in the motivation and academic decline of nursing students [22].

Based on the aforementioned information, although the model has yielded positive results, the responsibility of implementing it in clinical training lies with clinic coaches and faculty professors. Experts suggest involving clinical nurses in student education to ensure high-quality nursing education; however, nurses do not feel accountable for participating in the clinical education of students [20]. Therefore, considering the emphasis placed by experts on preparing clinical nurses to engage in nursing students’ education, this study was conducted to examine the impact of post-training on the involvement of clinical nurses in nursing students’ education.

## Materials and methods

A two-group-based quasi-experimental study with a pretest-posttest design was conducted in 2023. The research population included nurses from a selected educational hospital. The sample size was determined based on Heidari et al.’s study [23] using the mean comparison formula n = (Z_α/2_+Z_β_)^2^ *2*σ^2^ / d^2^, with a 95% confidence level, 80% test power, effect size equal 0.5 and accounting for a 10% sample loss, resulting in a sample size of 66 nurses.

The research units included nurses with a bachelor’s degree or above working in internal medicine, surgery, and emergency departments, where nursing students completed their internships. The participants also had at least one year of clinical work experience and were willing to participate in the study.

The exit criteria included not participating in half of the preceptorship training classes, transferring to departments without internship students, and withdrawing from the study.

Sampling Method: First, all nurses who met the criteria for inclusion in the study were selected as available and then randomly allocated to either the test or control group using a random block allocation method by the first author.

The data collection tool included a demographic characteristics questionnaire for nurses (age, sex, marital status, education level, place of residence, work experience, work sector, economic status, and overall time) and the IMSOC questionnaire (Questionnaire for assessing the participation of nursing professionals in the coaching of nursing students = International Maritime Staff Operators Course). The IMSOC questionnaire, designed by Cervera-Gasch, measures the level of participation of clinical nursing professionals in the education of students [24]. The questionnaire consists of five dimensions (implementation, motivation, satisfaction, obstacle, and commitment), for a total of 31 items. The questionnaire uses a five-point Likert scale (1: completely disagree; 2: disagree; 3: neither agree nor disagree; 4: agree; 5: completely agree). The minimum score on the questionnaire was 31, and the maximum score was 155. The intra-observer reliability was assessed using the intra-class correlation coefficient (ICC), with an ICC ≥ 0.75 indicating excellent agreement. The final version of the 31-item questionnaire had an internal correlation coefficient of 0.852 and excellent internal consistency, with a Cronbach’s alpha = 0.837.

The educational package was prepared and sent to a group of clinical education specialists based on existing notices and educational resources. Intervention involved various methods such as lectures, media, and linear training. The content covered the role model and the importance of the preceptor’s role in clinical education, the educational needs of the preceptor, readiness to accept the role and how to support students, the educational needs of the students, factors affecting the relationship between the student and the preceptor, problems faced by the preceptor, and how to implement and engage students in the wards. After the necessary corrections were made and the content was confirmed with experts, training workshops were conducted in two separate sessions, each lasting three hours and held one week apart, led by an expert professor in the field of clinical education.

At the beginning of the meeting, a questionnaire was completed and collected from the attendees. Simultaneously, with the workshop, 33 nurses assigned to the control group completed questionnaires.

After the workshop was held for four weeks and based on the content of the discussion, educational materials were presented on a social network, and 33 nurses were included in the intervention group. During this time, participants’ questions in the group were answered. Finally, after one month, the questionnaire was given again to the participants in both the intervention and control groups, and the data were collected after completion.

The control group did not receive any information. After completing the study, the educational content was presented to all nurses in the departments.

Data analysis was conducted using SPSS version 19 statistical software. Descriptive statistics such as prevalence, percentage, mean, and standard deviation were utilized to determine central (mean) and dispersion (standard deviation) indices. Inferential statistics, including the Kolmogorov-Smirnov test to check the normal distribution of data among groups, Levin’s F test to check the homogeneity of variance among groups, analysis of covariance test, paired t-test, and independent t-test, were also employed. A significance level of 0.05 was used.

## Results

A total of 66 nurses participated in the present study, all of whom remained through the end of the study. The research findings showed that the average age of nurses in the test group was 45.33 (7.43) years, while that of nurses in the control group was 48.15 (8.48) years. Furthermore, the average work experience of nurses in the test group was 16.90 (7.07) years, compared to 30.13 (8.57) years in the control group. The majority of nurses in the test group (51.5%) were male, while most nurses in the control group (63.6%) were female. There were no significant differences in terms of individual characteristics between the two groups (*P* > 0.05) (Table 1).

**Table 1:** Distribution of the absolute and relative frequency of research units based on demographic characteristics in two groups

Variable	Range	Intervention group		Control group		P value, test
		Number	Percent	Number	Percent	
Age (year)	30 − 20	2	1.6	12	4.36	P value: 0.08 t: 1.75
	40 − 31	8	2.24	14	4.42	
	50–41	13	4.39	3	1.9	
	Above 50	10	30.3	4	1.12	
Mean (SD)		45.33 ± 7.43		48.1 ± 8.48		
Work Experience	Mean(SD)	16.90 (7.07)		13.30 (8.57)		P value: 0.06 t: 1.86
Overtime (Hours)	Mean(SD)	1.27(0.45)		1.12(0.33)		P value: 0.12 t: 1.55
Sex	Male	17	51.5	12	36.4	P value: 0.32 χ٢ = 1.53
	Female	16	48.5	21	63.6	
Marital status	Single	4	12.1	7	21.2	P value: 0.33 ٢χ = 2.11
	Married	29	87.9	25	75.8	
	Divorced	0	0	1	3	
Education status	Bachelor	29	87.9	27	81.8	P value: 0.73 ٢χ 0.471
	Master degree	4	12.1	6	18.2	
Shift work	Morning	8	24.2	4	12.1	P value: 0.08 χ2 = 4.92
	Night	1	3	6	18.2	
	In circulation	24	72.2	23	69.7	
Employment status	Official	22	66.7	29	87.9	P value: 0.07 χ2 = 4.22
	Contract	11	33.3	4	12.1	
Income (million Rials)	Up to 100	7	21.2	2	6.1	P value: 0.23 χ2 = 3.31
	150 − 100	17	51.5	19	57.6	
	More than 150	9	27.3	12	36.4	
Department	Internal	14	42.4	19	57.6	P value: 0.08 χ2 = 5.39
	Surgery	10	30.3	12	36.4	
	Emergency	9	27.3	2	6.1	
Place of living	Tehran	29	87.9	32	97	P value: 0.35 χ2 = 1.94
	Others	4	12.1	1	3	
Number of children	0	11	33.3	7	21.2	P value: 0.10 χ2: 7.59
	1	11	33.33	5	15.2	
	2	7	21.2	10	30.3	
	3	4	12.1	9	27.3	
	4	0	0	2	6.1	

Standard deviation (SD)

**Fig. 1:**
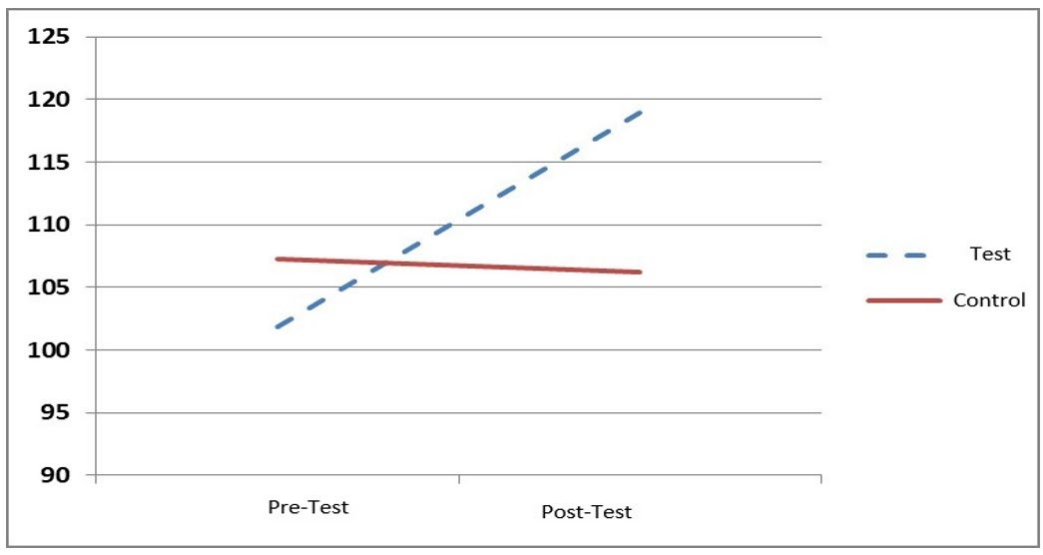
The average participation rate score change for all nurses studied

The average and deviation of the benchmark for the total participation of nurses in the education of students before intervention in the intervention group were 101.24 ± 10.42, and in the control group, were 107.84 ± 15.53. There were no significant differences based on the independent t-test (*P* = 0.10). After the intervention, the average participation score in the intervention group reached 118.90 ± 15.11, while in the control group, it reached 106.21 ± 11.96. The difference was significant according to the independent t-test (*P* < 0.001). A comparison of the mean scores and deviations of the overall participation scores in the intervention group using the t-test showed a significant difference from pretest to posttest scores, indicating an improvement in the overall nurses’ participation score (*P* < 0.001). However, in the control group, this difference was not significant from the pretest to the posttest (*P* = 0.41) (Table 2). The process of changing the average score of the participation rate of all nurses studied is displayed in Fig. 1.

**Table 2:** Comparison of the average participation and its dimensions in the units under study before and after intervention in two groups

Time dimension	Before	After	Result	
**Group**	Mean (SD)	Mean (SD)	*P value*	**Paired T-test
Intervention	26.66 (5.43)	29.6(3 5.54)	0.03	**-2.15**
Control	(6.04) 26.06	25.66(5.89)	0.42	0.80
*P-value	0.67	0.006		
*In depended T-test	0.42	2.81		
**Motivation**	Mean (SD)	Mean (SD)	P value	**Paired T-test
Intervention	22.33(4.37)	24.33(3.36)	0.03	**-2.27**
Control	23.78(3.83)	23.24(3.61)	0.09	**1.72**
*P value	0.15	0.20		
*In depended T-test	-1.43	1.26		
**Satisfaction**	Mean (SD)	Mean (SD)	*Pvalue*	**Paired T-test
Intervention	23.27(5.07)	29.00(4.50)	< 0.001	**-4.31**
Control	25.24(3.26)	25.12(3.95)	0.87	**0.16**
*P value	0.06	*P* < 0.001		
*In depended T-test	-1.87	3.71		
**Obstacle**	Mean (SD)	Mean (SD)	*P value*	**Paired T-test
Intervention	17.36(4.23)	21.72(4.44)	< 0.001	**-4.25**
Control	19.09(2.86)	18.81(3.72)	0.58	**0.54**
*P-value	0.057	0.005		
*In depended T-test	-1.94	2.88		
Commitment	Mean(SD)	Mean(SD)	*P value*	**Paired T-test
Intervention	12.21(2.50)	14.21(1.05)	< 0.001	**-4.30**
Control	13.06(1.83)	36.13(1.69)	0.23	**-1.22**
*P value	0.12	0.01		
*In depended T-test	-1.65	2.44		
**Total participation**	Mean (SD)	Mean (SD)	*P value*	**Paired T-test
Intervention	101.84(15.42)	118.90(15.11)	< 0.001	**-3.88**
Control	107.24(10.53)	106.21(11.96)	0.41	**0.82**
*P value	0.10	*P* < 0.001		
*In depended T-test	-1.65	3.78		

Standard deviation (SD), *Independent t test, ** paired t test.

## Discussion

The present study aimed to determine the effectiveness of a preceptorship training program for the participation of clinical nurses in nursing students’ education. The results indicated that the total participation score and all questionnaire dimensions were significantly improved in the intervention group. This finding is consistent with a previous study reporting the positive impact of a training course on the behavior of nurses in clinical education. The study also revealed that nurses working in public hospitals are significantly less involved in preceptorship educational programs. However, the study found an increase in only the dimensions of commitment and clinical education [25]. In our study, all dimensions of the questionnaire showed significant improvement. The qualitative study emphasized the significance of an effective teacher who can inspire students, employ innovative educational tools, and interact with students in a personal and approachable manner. Therefore, receiving training is one of the necessities of implementing the preceptor ship model [26].

The competence of coaches is an important topic that should be considered internationally when preparing the next generation of nurses. Therefore, preceptor ship training programs for coaches are necessary. Coaches around the world should be eligible for training and preparing nurses in the future [26].

A study showed that preceptor ship training is effective at improving the clinical knowledge of nurses. It is important to investigate the impact of this increased ability at the bedside [27]. Tayibi et al. 2020 conducted a study to examine the impact of utilizing experienced clinical nurses as clinical teaching assistants as a means to enhance the clinical skills of nursing students. The findings revealed that while this educational model effectively enhanced students’ ability to work independently and improved their functional skills through exposure to real-world environments, it also presented several challenges. These challenges need to be addressed through a collaborative approach to overcome existing obstacles and improve the interactions between the education and treatment teams. It is crucial to achieve the objectives of this educational model [16]. In another study, Wu et al. reported that preceptor training programs are beneficial. However, further research is recommended to explore the value and cost of having preceptors in educational settings [28]. Another study investigated the impact of the preceptorship program on self-efficacy and learning outcomes, and the results showed an increase in these areas [29]. In the present study, a perceptive intervention, which is an essential aspect of a nurses’ role, was utilized. This includes formally guiding nursing students in the department and informally assisting less experienced colleagues with their practical work. Nurses have many responsibilities toward students, and those considering this role should be familiar with the expectations and responsibilities associated with it [30].

One of the most important advantages of this study was the use of a questionnaire with good validity and reliability. This questionnaire specifically evaluated the participation of clinical nurses in the education of nursing students.

One limitation of this study was the use of a self-report questionnaire. There is a possibility that respondents misappropriated the completion of the self-report questionnaire, which can be considered a limitation of using this type of questionnaire.

It is suggested that professionals include extensive educational preparation and provide more educational and organizational support for the preceptor. Developing national standards for preceptor preparation and teaching techniques appropriate for students, as well as providing students with the opportunity to practice, will be effective in ensuring the success of this educational model. Other solutions provided include the commitment and willingness of students to provide direct patient care, the appointment of a preceptor throughout the entire clinical period, and the awareness of the student’s expectations and practical level. It is recommended in addition to the perceptors themselves, student should also evaluate the amount of participation of clinical nurses in training.

## Conclusion

The results and findings of this study demonstrated that the preceptor training program appeared to be effective in increasing the participation of clinical nurses in the education of nursing students. Given the importance of clinical training provided by nurses and the essential role of preceptors, it is recommended to implement preceptor training programs to enhance the participation of clinical nurses in the education of nursing students.

## Data Availability

The datasets generated and/or analysed during the current study are not publicly available due to its ethical concerns, supporting data cannot be made openly available, but are available from the corresponding author on reasonable request.
